# Evaluation of Different Techniques for Size Determination of Drug Nanocrystals: A Case Study of Celecoxib Nanocrystalline Solid Dispersion

**DOI:** 10.3390/pharmaceutics11100516

**Published:** 2019-10-07

**Authors:** Amanpreet Kaur, Prashantkumar Khodabhai Parmar, Arvind Kumar Bansal

**Affiliations:** Solid State Pharmaceutics Lab, Department of Pharmaceutics, National Institute of Pharmaceutical Education and Research (NIPER), Sector-67, S.A.S Nagar, Mohali, Punjab-160062, India; amanchahal027@gmail.com (A.K.); prashantpniper@gmail.com (P.K.P.)

**Keywords:** Celecoxib, Nanocrystalline Solid Dispersion, agglomerates, aggregates, discriminatory dissolution testing

## Abstract

Celecoxib (CEL) Nanocrystalline Solid Dispersion (CEL_NCSD) was generated by spray drying CEL, mannitol (MAN) and sodium lauryl sulfate (SLS) from a solvent mixture of methanol, acetone and water. The purpose of the work was to determine the size of CEL nanocrystals, investigate agglomeration and inspect dissolution of CEL_NCSD. Size determination was challenging as CEL nanocrystals are embedded in the matrix of MAN. Firstly, neat CEL_NCSD was analyzed using Scherrer equation. Secondly, MAN was dissolved in an aqueous stabilizer medium to selectively measure the size of CEL nanocrystals. Raman Spectra captured in Morphologi G3-ID confirmed the presence of CEL-only particles in the media. This dispersion gave D_90_ values of 882 ± 170.34 nm in Zetasizer. Discriminatory dissolution studies confirmed total release of 34.61 ± 1.59%, 47.42 ± 0.24%, and 44.61 ± 1.11% at 120 min from a microsuspension (size 3 µm), a nanosuspension (media milled; size 660 nm) and CEL_NCSD, respectively. The dissolution profile of CEL_NCSD was similar to that of a nanosuspension (f2 72.24) instead of a coarse microsuspension. Thus, the present study revealed that optimized sample preparation is critical for the size determination of embedded drug nanocrystals in NCSD. Further, a discriminatory dissolution study substantiated that the size of CEL nanocrystals in CEL_NCSD is well below 1000 nm, thus showing a size-dependent improved dissolution profile.

## 1. Introduction

Pharmaceutical nanocrystals are pure drug crystals, having at least one dimension between a few nanometers and 1000 nm (= 1 μm), and they exhibit size-dependent performance properties [1]. Nanocrystals enable high drug loading of up to 30%–40% in oral solid dosage forms [2,3]. They can be physically stabilized against agglomeration/aggregation by the use of stabilizers such as polymers and surfactants [4,5]. Nanocrystals are a promising strategy for improving apparent aqueous solubility and dissolution rate, leading to numerous biopharmaceutical advantages in the case of Biopharmaceutics Classification System (BCS) Class II/IV drugs [3,6,7].

The techniques for generation of drug nanocrystals fall into two categories: ‘bottom-up’ (solution to crystals) and ‘top-down’ (micro- to nanocrystals) approaches. Both the techniques generate a nanosuspension, which contains drug nanocrystals suspended in the liquid medium. [1,8,9,10,11]. Drying of “nanosuspensions” is then carried out using matrix-forming sugars, sugar alcohols or insoluble excipients such as microcrystalline cellulose to keep the nanocrystals segregated and enhance their long-term stability [12,13,14,15]. These techniques suffer from limitations related to the generation of agglomerates or aggregates of drug nanocrystals during drying, which can differentially impact their dissolution behavior [16,17].

**Figure 1:**
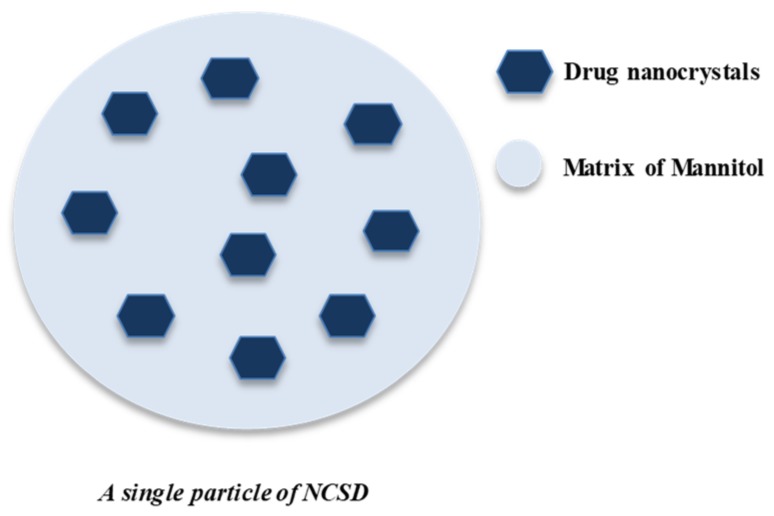
Pictorial representation of Celecoxib Nanocrystalline Solid Dispersion (CEL_NCSD) wherein CEL nanocrystals, stabilized using sodium lauryl sulfate (SLS), are embedded in the matrix of mannitol (MAN).

We have previously reported the generation of Nanocrystalline Solid Dispersion (NCSD) using a novel, one-step spray drying-based process named “NanoCrySP technology” [18]. It is a bottom-up process wherein the drug, crystallization-inducing excipient and stabilizer are spray dried from their solution. The excipient induces crystallization during spray drying by plasticization, heterogeneous nucleation and creating a physical barrier to crystal growth [19,20,21]. The salient feature of the technology is that a solid powder of drug nanocrystals is finally obtained. The size of primary particles of the matrix is in the range of 0.5 to 20 µm, consisting of drug nanocrystals (<1000 nm) dispersed in the matrix, as shown in Figure 1 [22,23].

Determination of the size of drug nanocrystals is relatively straightforward in the case of nanosuspensions generated using top-down and bottom-up approaches. In contrast, a nanocrystalline solid dispersion generated using NanoCrySP contains nanocrystals embedded in the matrix of the excipient. This poses significant challenges for the size determination of these embedded drug nanocrystals. Particle size determination of an unprocessed sample of NCSD would give the size of the primary particle containing the excipient matrix and drug nanocrystals. Another strategy could be to process the NCSD to form a suspension of drug nanocrystals for size determination. The contacts between the drug and excipient particles may need to be separated with the aid of mechanical processes, e.g., vortexing and bath ultrasonication.

Thus, the objective of the current work was to investigate different size determination techniques that allow for size determination of nanocrystals in NCSD with/without processing. NCSD of a poorly water-soluble drug, Celecoxib (CEL), was prepared using mannitol (MAN) and SLS as the crystallization-inducing excipient and stabilizer, respectively. A Powder X-Ray Diffraction (PXRD)-based Scherrer equation was used as the non-destructive method to determine the size of nanocrystals without processing of NCSD. Additionally, CEL_NCSD was processed to dissolve MAN and release CEL nanocrystals in an aqueous medium containing stabilizers, which was thereafter analyzed using Zetasizer. Morphologi G-3 ID was used to check the chemical identity of this suspension and to make sure that MAN dissolved in aqueous medium and CEL nanocrystals are released from the matrix. Orthogonal techniques such as SEM and TEM provided visual evidence of the size distribution of nanocrystals. The dissolution behavior of CEL_NCSD was compared to a nanosuspension of equivalent size and a microsuspension prepared by media milling to confirm the size-dependent increase in the dissolution performance of CEL_NCSD. The work highlights the specific information captured and limitations of different size determination techniques, and it is suggested to use multiple orthogonal techniques to obtain accurate results.

This work shall find applicability for nanosuspensions prepared using “top-down” and “bottom-up” methods, which are then dried in the presence of excipients for stabilization. In these cases, particle characterization is challenging, as nanocrystals have to be first separated from the excipient and information on particle size is mostly derived from indirect methods such as dissolution. A decision tree and steps for the characterization of the systems wherein nanocrystals are embedded in the matrix of an excipient have also been prepared.

## 2. Materials

Gift samples of Celecoxib (CEL), mannitol (MAN), SLS, Dioctyl Sodium Sulphosuccinate (DOSS) and Hydroxy Propyl Methyl Cellulose (HPMC) Low Viscosity E5 (HPMC LV E5) were received from Windlas Healthcare (Dehradun, India). Tri-sodium ortho phosphate dodecahydrate was purchased from Merck. Nylon syringe disc filters of size 13 mm and pore size 0.1 µm were purchased from MDI Technologies, Ambala, India. The organic solvents used—methanol, acetone, and hexane—were of analytical grade. All the compounds used were of purity ≥99%. Purified water generated in-house was used for all studies.

## 3. Methods

### 3.1. Generation of CEL_NCSD

CEL_NCSD was generated using NanoCrySP technology by slightly modifying the method described in our previous work [20]. A mixture of methanol: acetone: water in ratio 30:5:15 was selected for dissolving CEL, MAN and SLS in ratio 30:67.5:2.5, with a total solid content of 2% *w/v*. Accurately weighed CEL was first dissolved in methanol and acetone, whereas MAN and SLS were dissolved in water, followed by the mixing of the two solutions. The solution was dried using laboratory scale Spray Dryer (U228, Labultima, Mumbai, India) with a 2-way nozzle of diameter 0.7 mm, at 120 °C inlet temperature, 60 °C outlet temperature, 3 mL/min feed rate, 1.2 Kg/cm^2^ atomization pressure and 95–105 mm of Water Column (mmWC) of aspiration speed. A thermocouple (Thermo Scientific, USA) was mounted in the drying chamber to monitor the actual temperature achieved during drying. The spray dried product (CEL_NCSD) was collected and spread in a petriplate lined with aluminum foil. The petriplate containing CEL_NCSD was covered with aluminum foil and annealed at 60 °C for 24 h.

### 3.2. Crystallinity of CEL_NCSD

#### 3.2.1. Thermal Characterization Using Differential Scanning Calorimetry (DSC)

CEL_NCSD (2–3 mg) was weighed in a Tzero Aluminum pan, equilibrated at 40 °C and subjected to heating at the rate of 20 °C/min up to 200 °C using DSC (DSC Q2000, TA Instruments, New Castle, USA).

#### 3.2.2. Powder X-Ray Diffraction (PXRD)

The PXRD pattern of CEL_NCSD was recorded using a diffractometer (RigakuUltimia IV diffractometer, Tokyo, Japan). The X-ray generator was operated at 40 kV and 40 mA power settings. An approximate weight of CEL_NCSD ≈ 300 mg was loaded in a 25 mm poly- methyl methacrylate (PMMA) holder and gently pressed by a clean glass slide to ensure co-planarity of the powder surface with the surface of the holder. The pattern was recorded in a continuous scan mode with a step size of 0.01° and step time of 1 sec over an angular range of 3° to 40° 2θ.

### 3.3. Size Determination and Morphology of CEL_NCSD Primary Particles

The size of primary NCSD particles was determined by laser diffraction using a Malvern Mastersizer (Mastersizer 3000, Malvern PANalytical, Worcestershire, UK). CEL_NCSD was placed into the vibratory hopper of the dry dispersion unit and three consecutive repeat measurements were taken. The pressure employed was adjusted to 4 bars. The subsequent values of Dv10, Dv50 and Dv90 were noted down.

The morphology of CEL_NCSD was studied using Scanning Electron Microscopy (SEM) (S-3400, Hitachi Ltd., Tokyo, Japan) operated at an excitation voltage of 15 kV. CEL_NCSD powder was mounted onto a double-sided adhesive tape pasted over sample stubs and sputter-coated with gold using an ion sputter (E-1010, Hitachi Ltd., Tokyo, Japan) before analysis.

### 3.4. Size Determination of CEL Nanocrystals Embedded in CEL_NCSD Using Scherrer Equation without Dissolving the Matrix of MAN

The Scherrer equation was used to calculate the crystallite size of CEL from the PXRD pattern of CEL_NCSD and the physical mixture of CEL, MAN and SLS in ratio 30:67.5:2.5. The Scherrer equation (Equation (1)) depicts τ as the crystallite dimension, where K is the dimensionless numerical constant called the shape factor (0.94), λ is the wavelength (1.542 nm), and βt is the peak broadening due to size reduction measured as the full width half maxima (FWHM) of the PXRD peak. Characteristic diffraction peaks of CEL at 2θ values of 14.8°, 16.1° and 21.5° were used for crystallite size analysis. Peak full width at half maxima values for the characteristic peaks of CEL were determined in case of the physical mixture and CEL_NCSD. Size was calculated as the average of these three peaks [24]. The crystallite size was determined using Equation (1):

(1) τ=KλβtCosθ

### 3.5. High Performance Liquid Chromatography (HPLC) Method

The analytical method was developed for quantification of CEL using a HPLC system (Shimadzu, Japan) equipped with a column oven (CTO-10A vp), auto sampler (SIL-20 AC), Photodiode array detector (SPD- M20A), connector (CBM-20A), pump (LC-20AT), and LC solutions software system. The chromatographic conditions consisted of LiChrosphere ^®^100 symmetry RP-18 end capped (5 µm) run in isocratic mode using Acetonitrile and Phosphate Buffer (adjusted to pH 3) in ratio 70:30 at a flow rate of 1 mL/min. The injection volume of 20 µL was run for 10 min and was analyzed at 254 nm.

### 3.6. Size Determination of CEL Nanocrystals Embedded in CEL_NCSD by Dissolving the Matrix of MAN

#### 3.6.1. Selection of the Dispersion Medium

An aqueous medium was selected as the dispersion medium based on freely and poorly soluble nature of MAN and CEL in water (3 µg/mL), respectively. Stabilizers were added in water for steric stabilization of CEL nanocrystals in the medium. Stabilizers were chosen from reported commonly used stabilizers for the stabilization of nanocrystals. Also, a few of them have been reported in commercial products, e.g., SLS is present in innovator capsules of Celecoxib-Celebrex^®^. The stabilizer-based dispersion medium was finally screened based on contact angle study. Briefly, the contact angle of CEL was measured by the sessile drop method using a Drop Shape Analyzer instrument (FTA 1000, First Ten Angstrom, Virginia, USA). CEL were mounted on double-sided adhesive tape adhered to a glass slide followed by tapping to remove the excess powder, without compression. A drop of probe liquid (aqueous medium containing varying but below critical micelle concentration (cmc) % of a combination of surfactants and polymers, e.g., 0.1% *w/v* egg lecithin, 0.1% *w/v* Hydroxy Propyl Cellulose SSL, 0.1% *w/v* HPMC LV E5, 0.1% *w/v* Poloxamer 407, 0.1% *w/v* PVP K 30, 0.03% *w/v* SLS and 0.6% *w/v* DOSS) was dispensed separately onto the CEL surface, and video images were captured using an FTA image analyzer. The instrument calculated contact angle by mathematical fitting of the drop shape slope of the tangent to the drop at the liquid–solid–vapor interface. The dispersion of CEL_NCSD in the final surfactant-based medium was filtered through 0.1 µm nylon disc filters and analyzed using HPLC to rule out dissolution of CEL nanocrystals in the medium.

#### 3.6.2. Microscopic Imaging and Raman Analysis

CEL_NCSD (2.5 mg containing 0.75 mg CEL) was dispersed in an aqueous medium containing 0.1% *w/v* HPMC LV E5, 0.06% *w/v* DOSS and 0.03% *w/v* SLS in a 10 mL volumetric flask. The sample was then subjected to vortexing for 5 min followed by ultrasonication at 120 W and 40 KHz (Power sonic 510, Hwashin Technology, Seoul, Korea) for 10 min. This sample was then spread onto a 4.5*4.5 mm glass slide, air-dried followed by manual drying using a hand held hair dryer, and imaged in a Morphologi G3 microscope (Morphologi G3-ID, Malvern PANalytical, UK) at 20X. Morphologi G3-ID is an integrated system with a microscope and a Raman Spectroscope. The Raman system involves a RamanRxn1™ spectrometer from Kaiser Optical Systems, inc., United States. An input of approximate 50,000 particles was selected for automatic imaging by the microscope. A few particles were highlighted for Raman spectra performed using the integrated Raman spectrophotometer available in the Morphologi G3-ID instrument. The Raman spectrum for particles was collected in the range of 100–1825 cm^−1^. The excitation wavelength of the laser was 785 nm, time of exposure was 10 sec and resolution was 4 cm^−1^. Raman spectra of particles below 5 µm could not be captured as Morphologi G3-ID takes the Raman spectrum from a spot approx. 3 μm in diameter at the center of mass of the particle. Reference spectra of CEL and MAN were also captured for comparison. The spectra of particles of interest were compared with the reference spectra of CEL and MAN. Morphologi G3-ID does an automated calculation for determination of the chemical similarity of two compounds under the option “Raman correlation”. The scores of spectra were matched, and unity (=1) indicated identical spectra, while 0 indicated no resemblance with the reference spectra. The particle of interest was classified as pure CEL when the score of CEL was ≥0.75, and as MAN when the score was ≤0.45, and vice versa. The remaining particles were classified as aggregates of CEL and MAN and expected to contain peaks of both the components. The spectra of particles of interest were also compared visually with the reference spectra of CEL and MAN to confirm that no peaks of another component were observed.

#### 3.6.3. Optimization of Processing Parameters for Size Determination of CEL Nanocrystals Embedded in CEL_NCSD Using Zetasizer

A CEL_NCSD dispersion was prepared by dissolving 2.5 mg of CEL_NCSD in 10 mL of the stabilizer medium selected in Section 3.6.1. The size measurement showed variable data since the sample preparation was critical and the vortexing and sonication time affected separation of MAN from CEL nanocrystals as well as the separation of agglomerates. Therefore, processing and the measurement related variables were optimized. Ultrasonication breaks particle agglomerates due to the cavitation phenomenon. The variability in sonication treatment was optimized to enable reproducible size measurement of the nanocrystals. Hot spots in the bath tank were identified with an aluminum foil test. Active zones were marked where extensive erosion of foil occurred in 5 min [25]. The bath temperature was maintained at 24 ± 1 °C by replacing with fresh water twice a day and providing gaps between use of the instrument to ensure minimal heating and allowing water to cool down [25,26]. The sample was carefully transferred into a cuvette, avoiding transfer of bubbles/foam formed due to the presence of surfactant, and was analyzed immediately in a Malvern Zetasizer (Nano ZS, Malvern PANalytical, Worcestershire, UK) using disposable cuvettes at various parameters. A single measurement of five runs of 5 s each was taken in backscattering manual mode. The refractive index and absorption values for CEL were 1.6 and 0.010, respectively. The viscosity and refractive index of the dispersant medium were 1.28 cP and 1.330, respectively. The dispersion (10 mL) was analyzed to obtain six readings of a 1.5 mL sample each and the average of these six measurements was reported. The sample was analyzed within minutes to prevent nanocrystal aggregation.

### 3.7. SEM as an Orthogonal Technique for the Size Determination of CEL Nanocrystals Embedded in CEL_NCSD by Dissolving the Matrix of MAN

SEM (S-3400, Hitachi Ltd., Tokyo, Japan) was used as an orthogonal technique to corroborate the results of size obtained using Malvern Zetasizer. The dispersion prepared for analysis in the Zetasizer as mentioned in Section 3.6.3 was put onto a double-sided adhesive tape pasted over a sample stub using a pipette. The sample was air dried for 15 min, sputter-coated with gold using an ion sputter (E-1010, Hitachi Ltd., Tokyo, Japan) and analyzed in SEM.

### 3.8. Impact of the Size of Nanocrystals on Dissolution of CEL_NCSD

#### 3.8.1. Preparation of Microsuspension (MS_CEL) and Nanosuspension (NS_CEL) Using Wet Media Milling

MS_CEL and NS_CEL were prepared using the wet media milling technique. CEL (150 mg, 5.0% w/w) was dispersed in 2.85 mL of aqueous stabilizer solution (0.1% *w/v* SLS and 1.0% *w/v* HPMC LV E5) in a vial and stirred at 400 rpm for 5 min using a magnetic stirrer. Glass beads weighing 8.0 g with dimensions of 0.2–0.3 mm were added to the coarse dispersion of CEL and stirred at 1000 rpm for 6 hrs. A 20 µL aliquot of the MS_CEL and NS_CEL was diluted up to 10 mL with filtered (0.1 μm) purified water for size determination using optical microscopy (Leica Microsystems Wetzlar GmbH, Germany) and the Malvern Zetasizer. The samples were vortexed and ultrasonicated at 40 Hz for 5 min each. The sample analysis parameters were the same as those mentioned in Section 3.6.3.

#### 3.8.2. Comparative Dissolution of MS_CEL, NS_CEL and CEL_NCSD Using Discriminatory Dissolution Medium

Dissolution was carried out using USP type II apparatus operated at 37 ± 0.5 °C and a paddle speed of 50 rpm. The dissolution medium comprising of 0.04 mM tribasic sodium phosphate buffer of pH 11.7 was allowed to reach the adjusted temperature. MS_CEL, NS_CEL and CEL_NCSD equivalent to 200 mg CEL were added into 1000 mL dissolution medium. Samples (2 mL) were collected at 5, 10, 15, 20, 30, 60 and 120 min and filtered through 0.1 μm syringe filters. An equal volume of blank medium maintained at 37 °C was replenished each time. The quantity of dissolved CEL was determined using the HPLC method, and % drug release with time was plotted. The chromatographic conditions for analysis of the sample are detailed in Section 3.5. The similarity (f2) factor was calculated for comparison of the dissolution profiles.

### 3.9. TEM Analysis of CEL Nanocrystals Embedded in CEL_NCSD

TEM (FEI TF-20; FEI, Hillsboro, Oregon) analysis was carried out to further confirm the particle size of CEL nanocrystals. The sample for TEM analysis was prepared by dispersing 2.5 mg of CEL-NCSD powder in 10 mL dispersant medium containing 0.1% *w/v* HPMC LV E5, 0.06% *w/v* DOSS and 0.03% *w/v* SLS. The sample was vortexed for 5 min to dissolve MAN and release CEL nanocrystals into the medium, without sonication. A drop of this sample was placed onto a carbon coated copper grid, air-dried at room temperature and analyzed under TEM at 200 kV.

## 4. Results

### 4.1. Generation of CEL_NCSD

The CEL_NCSD generated using spray drying was a free-flowing powder, which was partially crystalline and had amorphous content. CEL_NCSD was annealed at 60 °C, which is close to the glass transition (*T*g) of CEL (58 °C) to convert amorphous CEL to crystalline form. Molecular mobility in the amorphous form increases around *T*g and thus encourages crystallization [19]. The mechanism of generation of CEL nanocrystals by heterogeneous nucleation in the presence of MAN has been reported in our previous work [20].

### 4.2. Crystallinity of CEL_NCSD

#### 4.2.1. Thermal Characterization Using DSC

**Figure 2:**
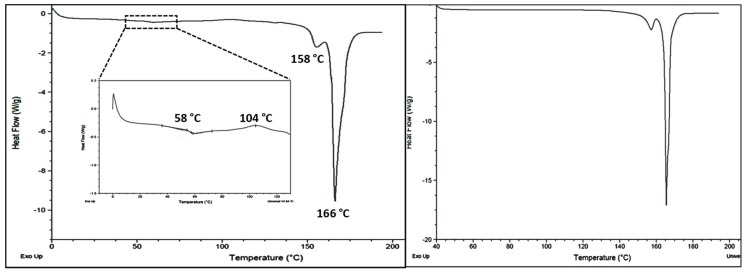
Differential scanning calorimetry (DSC) of unannealed and annealed CEL_NCSD samples. The unannealed sample showed the glass transition (*T*g) of CEL at 58 °C followed by recrystallization temperature (*T*c) at 104 °C. Thereafter, the melting of CEL and Mannitol at 158 °C and 166 °C was observed, respectively. The annealed sample showed the melting of CEL at 158 °C and that of Mannitol at 166 °C.

The DSC heating curve of annealed CEL-NCSD is depicted in Figure 2. The sample of CEL_NCSD showed sharp melting at 156.64 ± 2.728 °C (ΔH_f_ 8.95 ± 2.08 J/g) and 165.47 ± 2.02 °C (ΔH_f_ 166.45 ± 2.05 J/g) corresponding to the melting of CEL and MAN, respectively. These melting endotherms matched with stable Form III and Form α of CEL and MAN, respectively [27,28,29]. Since the melting points of polymorphic forms of MAN are close (β = 166.5 °C and α = 166 °C) and the metastable form δ converts to stable form during heating, the same could not be distinguished in DSC curves. PXRD provided more substantial evidence on polymorphic forms of CEL and MAN.

#### 4.2.2. PXRD

**Figure 3:**
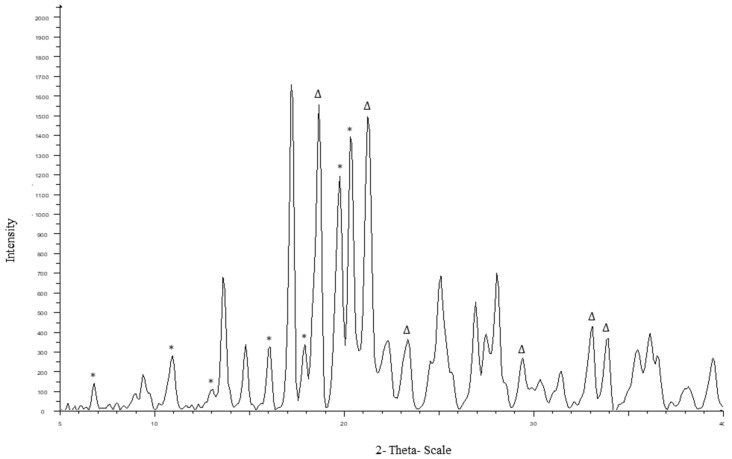
Powder X-ray diffraction (PXRD) pattern of CEL_NCSD showing characteristic peaks of CEL and MAN. Peaks for CEL and MAN are highlighted in * and ∆, respectively.

The diffraction pattern of CEL_NCSD is shown in Figure 3. The characteristic peaks of CEL were found at 2θ values of 5.32°, 10.7°, 12.9°, 14.9°, 16.1°, 19.7° and 21.5° and matched Form III of CEL. The specific peaks of MAN were observed at 18.6°, 21.8°, 24.5°, 29.7°, 33.2° and 33.7° and matched to a mixture of Forms α, β and δ [27,28,29].

### 4.3. Size Determination and Morphology of CEL_NCSD Primary Particles

The average particle size based on a volume fraction by laser diffraction using Mastersizer showed Dv_10_, Dv_50_ and Dv_90_ values of 0.876 µm, 7.504 µm and 16.439 µm, respectively. The specific surface area of CEL_NCSD particles calculated in Mastersizer was 2.797 m^2^/g.

**Figure 4:**
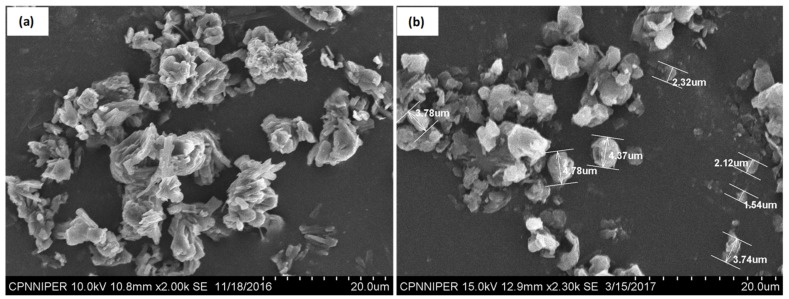
SEM images of primary particles of CEL_NCSD: (**a**) Clumps of needle-shaped CEL_NCSD particles; (**b**) Size measurement of CEL_NCSD particles.

The SEM images of primary particles are depicted in Figure 4. The needle-shaped particles were clumped together to form dense irregularly shaped agglomerates (Figure 4). Both CEL and MAN are reported to form needle-shaped and prismatic rod-shaped crystals, and similar morphological characteristics were observed in NCSD [28]. The size of the majority of particles as seen in SEM was between 0.5 and 9.8 µm. Powder was observed to exist as aggregates of the primary particles. The difference in particle size obtained in SEM and Mastersizer could be due to the presence of aggregates, which were excluded from visual measurement in the microscope but were measured by the laser diffraction using Mastersizer.

### 4.4. Size Determination of CEL Nanocrystals Embedded in CEL_NCSD Using Scherrer Equation without Dissolving the Matrix of MAN

**Figure 5:**
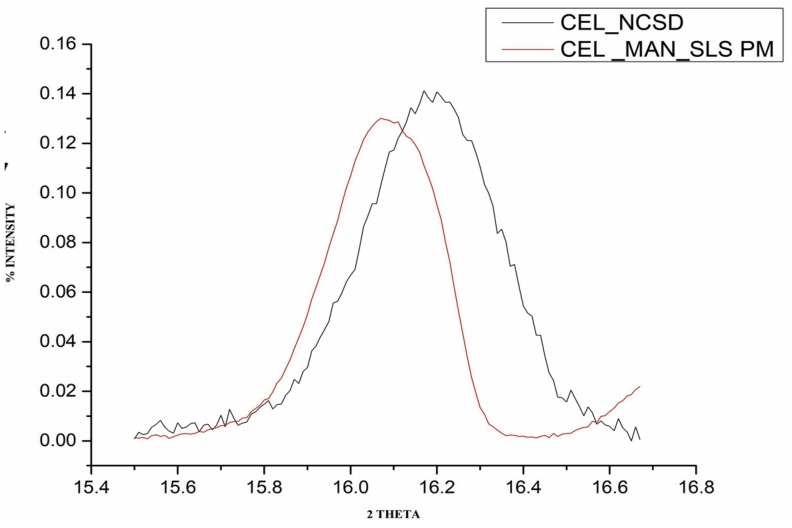
A representative image showing overlay of characteristic peaks at 2θ 16.1° ± 0.2 for (1) the physical mixture of CEL_MAN_SLS (30:67.5:2.5) and (2) CEL_NCSD.

The FWHM values for the physical mixture and CEL_NCSD are given in Table 1. Peak broadening observed in the case of nanocrystals is depicted in Figure 5. The size of CEL nanocrystals as determined using the Scherrer equation was found to be around 150 nm. The Scherrer equation allows size determination of the individual crystals without the need for sample processing.

### 4.5. Size Determination of CEL Nanocrystals Embedded in CEL_NCSD by Dissolving the Matrix of MAN

#### 4.5.1. Selection of the Dispersion Medium

Initially, water was selected as the dispersion medium, but the wetting of NCSD powder was poor due to the hydrophobicity contributed by CEL [30]. Thus, an aqueous dispersant medium containing a combination of non-ionic and polymeric stabilizers was selected based on contact angle studies. This allowed assessment of wetting as well as stabilization of CEL nanocrystals released from CEL_NCSD. These ultrafine particles have the tendency to form loose agglomerates to high surface free energy contributed by smaller size [13]. Selection of the dispersion medium was made based on contact angle as a lower contact angle indicates better wettability. The results of the contact angle of probe liquids with CEL are mentioned in Table 2. The minimum contact angle was found with 0.06% *w/v* DOSS solution. Amongst the combination systems, contact angle was in the following order: 0.06% *w/v* DOSS + 0.03% *w/v* SLS + 0.1% *w/v* HPMC LV E5 < 0.06% *w/v* DOSS + 0.1% *w/v* HPMC LV E5 < 0.1% *w/v* Poloxamer 407 + 0.06% *w/v* DOSS < 0.06% *w/v* DOSS + 0.1% *w/v* HPC SSL. The medium having the combination 0.06% *w/v* DOSS + 0.03% *w/v* SLS + 0.1% *w/v* HPMC LV E5 was selected based on the minimum contact angle. The concentration of the stabilizers used was well below their critical micelle concentration (CMC) and thus would not cause micellar solubilization of released nanocrystals in the dispersion medium [31]. Additionally, the amount of CEL dissolved in the dispersion medium was quantified to rule out significant solubility of CEL in the same. The assay of CEL was found to be ≤0.6%, thus ruling out significant dissolution in the dispersant medium.

#### 4.5.2. Microscopic Imaging and Raman Analysis

**Figure 6:**
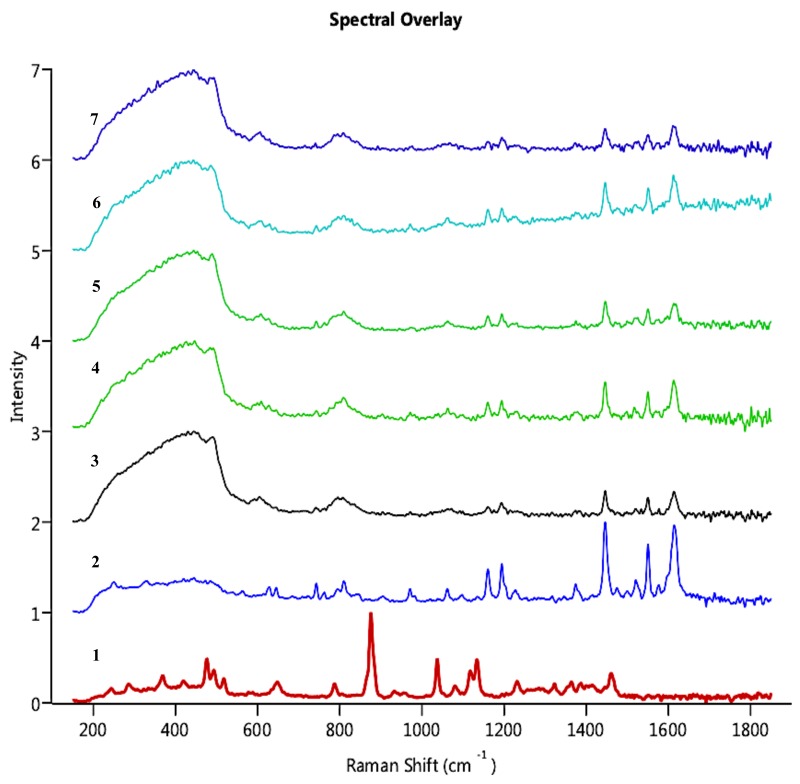
Overlay of Raman Spectra captured for particles in CEL_NCSD dispersion; the Spectra have been compared for (1) MAN, (2) CEL and selected particles in the dispersion (3) Particle 587, (4) Particle 9137, (5) Particle 23,642, (6) Particle 23,884 and (7) Particle 30,630.

Morphologi G3-ID gave information on the chemical nature of the suspension prepared by dissolving MAN contained in the NCSD. It was imperative to ensure that MAN dissolved completely to release CEL nanocrystals in the medium. As can be observed in Figure 6, the particles of interest showed characteristic peaks of CEL at 1150 cm^−1^, 1200 cm^−1^, 1440 cm^−1^, 1570 cm^−1^ and 1620 cm^−1^. As the score values of these particles were more than 0.75 except particle 30,630, the particles were identified to be of CEL. This confirmed the complete dissolution of MAN in the dispersion medium and the release of CEL nanocrystals from CEL_NCSD. The Raman correlation score values of selected particles are given in Table 3. Visual analysis also confirmed that the particles in the dispersion medium were of CEL as spectra of selected particles matched with pure CEL. The score value for particle 30,630 was 0.671, which was less than 0.75, but visual analysis showed that the spectrum was suppressed but matched with CEL.

Sample preparation for Morphologi G3-ID involved the drying of a drop of suspension on a glass slide. The resolution was only enough to visualize and measure nanocrystals as tiny dots. Also, drying of the sample led to particle aggregation as the solvent evaporated. Hence, these particles were not counted for size measurement. Evidence in support of aggregates present in the neat sample or formed during drying was generated by performing discriminatory dissolution testing and comparing the release profile of CEL_NCSD dispersion to micronized and nanonized suspensions of CEL.

#### 4.5.3. Optimization of Processing Parameters for Size Determination of CEL Nanocrystals Embedded in CEL_NCSD Using Zetasizer

Mere dispersion of CEL_NCSD in aqueous medium was insufficient to dissolve MAN and release CEL nanocrystals. Instead, the processing steps of vortexing and ultrasonication were critical for releasing CEL nanocrystals and forming a fine dispersion. The size measurements for dispersion are listed in Table 4.

*Vortexing:* The time for vortexing was fixed to 5 min, as further increase in vortexing time did not result in disruption of CEL_NCSD particles. Vortexing partially aids in the dispersion of CEL_NCSD by dissolving MAN into the aqueous medium, as was evident by higher values of Z_avg_, D_90_ and PDI and the presence of sediment at the bottom of the flask.

*Ultrasonication:* Vortexing for 5 min followed by variable ultrasonication times produced a relatively homogenous dispersion with fine particle size. Ultrasonication demonstrated time-dependent reduction in particle size till 10 min, after which no further size reduction was observed. This observation suggested that bath ultrasonication did not lead to size reduction of nanocrystals, but disrupts drug excipient bridges and agglomerates. A kilo counts per second (kcps) value between 200 and 400 is desirable to ensure optimal dilution of the sample. The kcps values were lower for samples processed by vortexing/sonication for 5/5 min, 5/15 min and 5/20 min. This indicated that either sample had not been processed properly or the particles were aggregates and/or sedimenting. At lower kcps values, D_90_ represents the size of a few small particles present in the supernatant. This was confirmed visually, as the particles settled in the volumetric flask at 5 min of sonication. The higher PDI values indicated the inhomogeneous nature of the dispersion. At 10, 15 and 20 min of sonication, although there was no apparent sedimentation, the Z_avg_ and D_90_ values of dispersion were ≈1 µm.

The time for ultrasonication was thus optimized to 10 min to produce fine dispersion. Visual analysis of samples showed ultra-fine particles with smoky appearance, and the diameter of particles could not be marked with the naked eye. This observation was used as the visual marker to ensure that sample preparation was done properly. The Z_avg_, D_90_, PDI and kcps values were found out to be 875 ± 160.94, 874 ± 170.34, 0.34 ± 0.22 and 275.33 ± 11.49 nm, respectively. The sample was analyzed within minutes to prevent nanocrystal aggregation. The further characterization of nanocrystals was carried out using SEM and TEM.

### 4.6. SEM as an Orthogonal Technique for the Size Determination of CEL Nanocrystals Embedded in CEL_NCSD by Dissolving the Matrix of MAN

**Figure 7:**
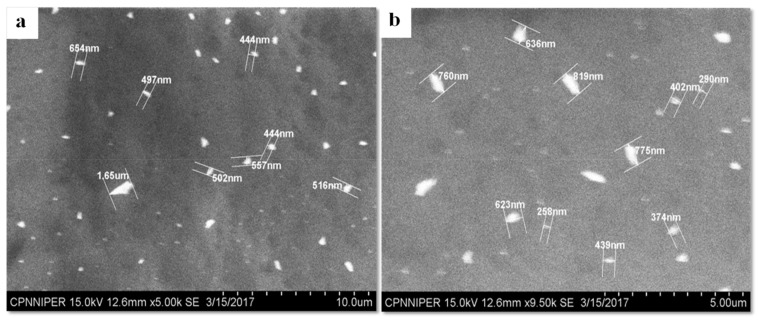
SEM images of CEL_NCSD dispersion. (**a**) and (**b**) images show CEL nanocrystals below 1000 nm.

The analysis of CEL_NCSD dispersion using SEM substantiated the particle size data, as can be seen in Figure 7. The majority of particles were well below 1000 nm. The images provided evidence of the presence of discrete particles, with no evidence of agglomerates or aggregates of nanocrystals. SEM, thus, corroborated the results collected in Zetasizer.

### 4.7. Impact of the Size of Nanocrystals on Dissolution of CEL_NCSD

#### 4.7.1. Preparation of MS_CEL and NS_CEL Using Media Milling

A coarse microsuspension (MS_CEL) and fine nanosuspension (NS_CEL) were prepared of sizes 3.5 ± 0.45 µm and 660 ± 210 nm to compare dissolution with CEL_NCSD. MS_CEL was obtained after milling for 3 h; however, NS_CEL was prepared by milling CEL for 6 h. NS_CEL was processed using vortexing and bath ultrasonication for size determination. This was due to the inherent tendency of nanocrystals to aggregate due to high surface free energy. The CEL_NCSD dispersion prepared in water, at the time of dissolution, showed a size of 787 ± 167 µm.

#### 4.7.2. Comparative Dissolution of MS_CEL, NS_CEL and CEL_NCSD Using Discriminatory Dissolution Medium

**Figure 8:**
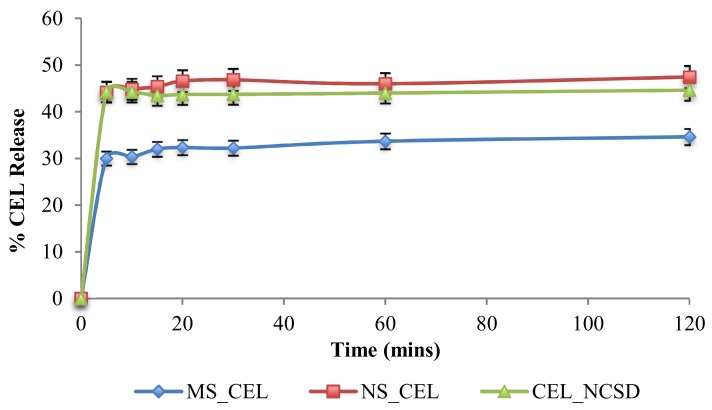
Percentage release profile of MS_CEL, NS_CEL and CEL_NCSD. The values are reported as Mean ± SD.

The percent release of CEL from MS_CEL, NS_CEL and CEL_NCSD is shown in Figure 8. CEL nanocrystals dissolved rapidly in almost 5 min from NS_CEL and CEL_NCSD, and the % CEL release was more or less constant at subsequent time intervals. The dissolution medium was non-sink and supported a solubility of 165.7 ± 11.8 µg/mL of CEL. Because of the non-sink nature, the solubilization capacity of the medium was limited. However, the medium provided discrimination in % CEL release from MS_CEL and NS_CEL.

CEL_NCSD performed better than the coarse microsuspension (f2 45.62), with 1.33 times higher CEL release. Since the dissolution profile of CEL_NCSD was equivalent to NS_CEL of D_90_ at 660 nm (f2 72.24), this confirmed that nanocrystals in CEL_NCSD were discrete as in NS_CEL. If CEL nanocrystals were released as agglomerates, these were loosely held and did not retard dissolution. It has been reported that the majority of the surface area is available for dissolution in loose agglomerates [12]. This supports the earlier hypothesis that the agglomerates observed in Morphologi G3-ID were formed during sample preparation. The study served as a surrogate for indirect size determination by comparison of dissolution profiles. The dissolution of CEL_NCSD was similar to a nanosuspension of size ≈600nm, thus confirming that nanocrystals in CEL_NCSD are discrete and of similar size.

### 4.8. TEM Analysis of CEL Nanocrystals Embedded in CEL_NCSD

**Figure 9:**
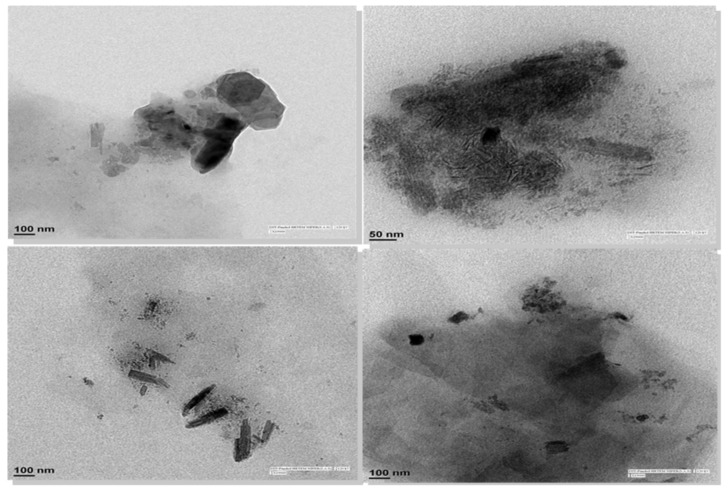
TEM images of CEL_NCSD dispersion showing CEL nanocrystals (dark spots) in the matrix of MAN (greyish black background).

The TEM images as shown in Figure 9 clearly show the presence of discrete nanocrystals (dark spots) embedded in the matrix of dissolved MAN (greyish black background), and loose agglomerates were present at certain sites. The size of these crystals was in the range of ≈ 50–300 nm. It is pertinent that the sample for TEM was prepared using vortexing and without any sonication. Hence, TEM provided direct evidence of the presence of discrete nanocrystals and loose agglomerates in the matrix of MAN in the case of CEL_NCSD.

## 5. Discussion

Size determination of nanocrystals in NCSD is challenging because of the nature and construction of the particles. Nanocrystals are embedded in the matrix of an excipient, hence two methodologies were employed: (i) analysis of the neat sample without extraction of nanocrystals, and (ii) nanocrystals were extracted from the matrix by dissolving it in a suitable medium. The Scherrer equation offered a tool to determine the size of crystallites without the need for extraction, and the size obtained for CEL nanocrystals in CEL_NCSD was close to 200 nm. It is pertinent to note that the Scherrer equation would provide the size of individual crystallites even when they are part of a polycrystalline sample. This may result in underestimation of the “practical” size of the nanocrystals.

The sample preparation protocol was optimized for the extraction of nanocrystals from the water-soluble matrix of MAN. Various parameters, such as the solvent to dissolve the excipient, stabilization of nanocrystals using stabilizers in the solvent, processing using vortexing and disruption of particle bridges and agglomerates using sonication, were optimized.

Morphologi G3-ID, comprising of Raman spectroscopy coupled with microscopy, is a new tool developed to simultaneously characterize the size and identity of particles. Raman spectra captured for CEL_NCSD dispersion established the identity of CEL and the absence of MAN from the particles. This indicated that MAN dissolved completely on processing and CEL-only nanocrystals remained suspended in the medium. This allowed selective measurement of the size of CEL nanocrystals from the dispersion. However, data on the particle size of nanocrystals obtained using Morphologi G3-ID were misleading due to the agglomeration of nanocrystals into clusters or chains during the drying step involved in sample preparation for analysis.

Subsequent analysis of this sample using Zetasizer and SEM provided comparable sizes of 787 nm and 550 nm, respectively. TEM visualizes a very limited area in the picometer’s range and demonstrated a size close to the Scherrer equation. However, both SEM and TEM provided clear visual evidence of the nanometer size of CEL nanocrystals.

Dissolution of CEL_NCSD was compared to a microsuspension (particle size D90, 3.5 µm) and a nanosuspension (particle size D90, 687 nm). A discriminatory dissolution medium was used to differentiate performance of micronized and nanonized samples. Dissolution is directly proportional to size and surface area as described by the Noyes–Whitney equation. The dissolution behavior of CEL_NCSD was similar to the nanosuspension of size 687 nm, in contrast to the microsuspension. The sample preparation protocol as optimized for Zetasizer was hence considered optimal, and the nanocrystals had a size in the nanometer range.

**Figure 10:**
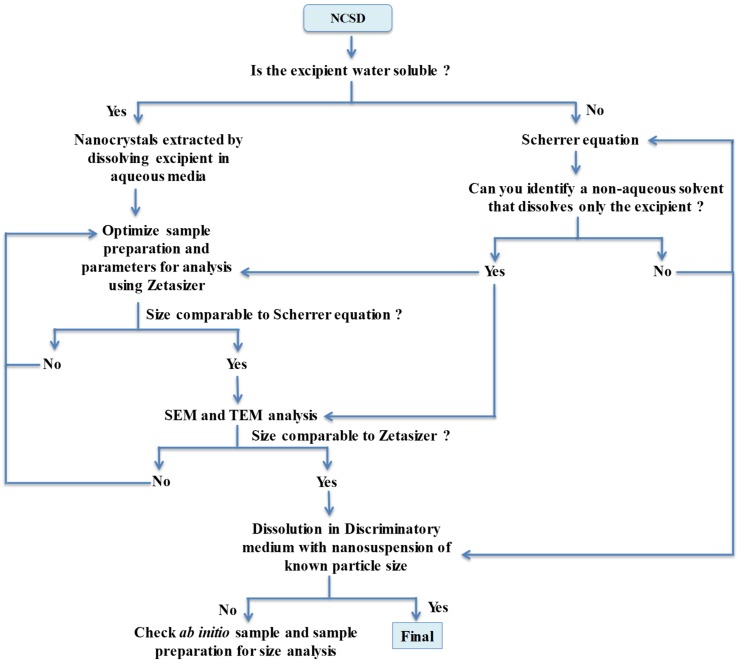
Decision tree for particle size determination of nanocrystals in NCSD.

The summary of information captured and limitations of various size determination methods are compiled in Table 5. A detailed flow chart and steps for particle size determination of nanocrystals are described in Figure 10. This work has utility for the development of nanocrystal-based products using “top-down” or “bottom-up” approaches. Orthogonal techniques are necessary to generate evidence for particle size without interference from other excipients of the nanosuspension or the finished drug product. The Scherrer equation can provide ab initio information without interference from other excipients, although the size is underestimated. A sound sample preparation protocol is imperative for meaningful data collection using Zetasizer and can serve as a routine standard testing procedure in an industrial setup. Use of additional tools like Morphologi G3-ID, SEM and TEM during the development stage would provide irrefutable evidence of the identity and size of nanocrystals.

## 6. Conclusions

Nanocrystalline Solid Dispersion generates nanocrystals embedded in the matrix of the excipient, thus making their characterization challenging. The Scherrer equation provided an ab initio tool to quantify the size of nanocrystals. However, this method would not differentiate the aggregates of nanocrystals with fused grain boundaries. The optimization of vortexing and ultrasonication allowed determination of particle size distribution by Zetasizer. The same sample was characterized by SEM, which provided supporting data for particle size. Morphologi G3-ID provided insights into the chemical nature of particles based on Raman Spectra and confirmed the presence of CEL-only particles. Similar dissolution profiles of CEL_NCSD and CEL_NS were obtained in discriminatory media, thus establishing the validity of the particle size determined using Zetasizer. It was further established that vortexing/sonication did not cause size reduction, thus contributing to erroneously small size. TEM provided clinching evidence on the presence of predominantly discrete nanocrystals with a few loose agglomerates.

This work provides an experimental framework for the determination of particle size in Nanocrystalline Solid Dispersion prepared using a novel platform technology—NanoCrySP. Additionally, the work has broader implications for types of systems currently exploited in drug delivery and manufacturing. It would provide deeper insights into methods of size determination and particle characterization in dried nanosuspensions, finished nanocrystal-based products, co-encapsulated systems, Trojan microparticles, admixtures of co-processed active pharmaceutical ingredients and excipients. Spray dried admixtures have been increasingly utilized for continuous manufacturing, where size determination of active pharmaceutical ingredients (APIs) become challenging. This work provides an understanding of critical material attributes, such as particle size, necessary to achieve critical quality attributes, e.g., dissolution.

## Figures and Tables

**Table 1:** Peak full width at half maxima (FWHM) values and peak broadening of PXRD peaks of CEL crystals in the physical mixture and CEL_NCSD.

2θ (°)	FWHM		Peak Broadening	Crystallite Size (nm)
	Physical Mixture	CEL_NCSD		
14.8	0.358	0.407	0.049	163.56
16.1	0.294	0.33	0.036	223.66
21.5	0.375	0.452	0.076	106.24
Average Size ± SD				164.48 ± 58.71

**Table 2:** Contact angle values of various stabilizer solutions with CEL (the values are the average of three measurements).

Stabilizers (% *w/v*)	Contact Angle Values (θ) (Mean ± SD)
0.1% egg lecithin	121.66 ± 0.57
0.1% HPC SSL	103.33 ± 1.52
0.1% HPMC LV E5	112.66 ± 4.50
0.1% Poloxamer 407	105.33 ± 1.52
0.1% PVP K 30	118.66 ± 4.16
0.03% SLS	114.66 ± 1.52
0.06% DOSS	70.33 ± 0.57
0.06% DOSS + 0.03% SLS + 0.1% HPMC LV E5	48.33 ± 2.08
0.06% DOSS +0.1% HPMC LV E5	52.00 ± 3.46
0.06% DOSS + 0.1% HPC SSL	61.00 ± 1.00
0.1% Poloxamer 407 + 0.06% DOSS	54.66 ± 2.08

**Table 3:** Raman Correlation Scores for selected particles/particles of interest in the dispersion.

Particles of Interest	Raman Correlation Score (with Pure CEL)	Remarks
Particle 587	0.723	Spectra were found similar to CEL
Particle 9137	0.855	
Particle 23,642	0.713	
Particle 23,884	0.834	
Particle 30,630	0.671	

**Table 4:** Impact of critical process parameters of CEL_NCSD for size determination of embedded nanocrystals of CEL using Zetasizer. The values are the average of six measurements (Mean ± SD).

Processing Parameters		Zetasizer Parameters			
Steps	Time (mins)	Z_avg_ (nm)	D_90_ (nm)	PDI	kcps
Vortexing	5	2414 ± 358.2	402 ± 0.217	0.393 ± 0.05	205.60 ± 12.43
Vortexing/Ultrasonication	5/5	1798 ± 176.99	666 ± 64.08	1.00 ± 0.00	167.96 ± 37.62
Vortexing/Ultrasonication	5/10	1255 ± 160.74	574 ± 103.40	1.00 ± 0.00	250.00 ± 114.60
Vortexing/Ultrasonication	5/15	1490 ± 429.96	452 ± 55.62	0.921 ± 0.08	219.13 ± 32.88
Vortexing/Ultrasonication	5/20	1455 ± 160.74	274 ± 103.40	1.00 ± 0.00	143.13 ± 12.02

**Table 5:** Summary of information captured and limitations of various size determination methods.

S.No.	Techniques	Information Captured	Comments
1.	Scherrer equation using PXRD	Size of ab initio sample	Cannot distinguish between discrete and agglomerated particles
2.	Vortexing and Ultrasonication	Sonication helps in the formation of a nanosuspension	Chances of size reduction and/or aggregation by sonication
3.	Zetasizer	Size of particles below 1000 nm	Significant effort required for optimization of the method
4.	SEM	Size of particles below 1000 nm	Size may not be representative of the whole sample
5.	Morphologi G3-ID	CEL observed as discrete particles and agglomerates	Aggregation during drying of the sample
6.	Discriminatory dissolution	Dissolution of CEL_NCSD was similar to NS_CEL	Useful tool for establishing performance criteria of nanocrystals
7.	TEM	Discrete CEL nanocrystals in the matrix of MAN	Sample size may not be representative of the whole sample
